# Polymerase chain reaction of enterococcus faecalis and candida albicans in apical periodontitis from Turkish patients

**DOI:** 10.4317/jced.50669

**Published:** 2012-02-01

**Authors:** Aysin Dumani, Oguz Yoldas, Sehnaz Yilmaz, Fatih Koksal, Begum Kayar, Beril Akcimen, Gulsah Seydaoglu

**Affiliations:** 1 DDS, PhD. Assistant Professor, Cukurova University, Faculty of Dentistry, Department of Endodontics; 2 DDS, PhD. Professor, Cukurova University, Faculty of Dentistry, Department of Endodontics; 3 MD, PhD. Professor, Cukurova University, Department of Microbiology; 4 DDS, PhD. Research Asistant, Cukurova University, Department of Microbiology; 5 MD, PhD. Associated Professor, Cukurova University, Department of Biostatistic

## Abstract

Aim: The aim of this study was to determine the frequency of two important pathogenic microorganisms associated with endodontic infections, Enterococcus faecalis and Candida albicans, in root canal samples from patients with necrotic pulps or failed canal therapy by polymerase chain reaction method. Method: Microbial samples were obtained from 117 teeth with necrotic pulp tissues and 114 teeth with failed endodontic treatment. Results: E.faecalis were identified in 16% of the necrotic and 10% of the retreated root canal infections by PCR. C.albicans genome were identified in 20% and 11% of the necrotic and retreated root canal infections, respectively, by PCR. The frequencies of microbiota were not statistically different between necrotic and retreatment groups (p > 0.05, chi squared test). Conclusions: PCR analysis of teeth with periapical lesions revealed that E.faecalis was found in fewer patients than in previous studies. The C.albicans prevelance was consistent with previous reports. No statistical difference was found between primary and secondary root canal infections for C.albicans or E.faecalis.

** Key words:**Primary root canal infection, secondary root canal infection, E.faecalis, C.albicans.

## Introduction

The most important factor associated with endodontic failure is the presence of persistent microbial infection in the root canal system (1). In most studies, the root canal microbiota of teeth with failed endodontic treatment is different from the flora found in untreated teeth (2-4).

Necrotic root canals are typically polymicrobial, with nearly equal proportions of Grampositive and Gramnegative bacteria, and are dominated by anaerobic bacteria. Additionally, anaerobes are, in many cases, accompanied by microaerophilic and facultative bacteria (5). In contrast, the microbial flora in retreatments have been described as monoinfections or infections including a few Grampositive bacterial species, with approximately equal proportions of facultative and obligate anaerobes (4). Root-filled teeth with apical periodontitis microbiota contain *Enterococcus* spp. together with *Streptococci, Lactobacilli*, facultative bacteria, and anaerobic bacteria. Gram-negative enteric rods and yeasts have also been found in retreatment cases (4,6).

In recent microbiological studies, increasing concern has arisen about *Candida albicans (C.albicans)* and *Enterococcus faecalis (E.faecalis)* in failed root canal treatments. *E.faecalis* can occur in primary root canal infections, especially in teeth with coronal leakage, although typically in low numbers (7) and also is the most common organism cultured from failed root canal therapy, with 12–90% prevalence (8,9). This disparity in the composition of root canal microbiota can be related to differences in methodologies of studies, but may also be influenced by other factors, such as geographical effects (10). Bacteria may originate in the oral cavity and contaminate the root canal during treatment due to inadequate aseptic control or penetrate the root filling via coronal leakage after root canal treatment (11). Engström (12) found a direct relationship between the occurrence of enterococci in the oral cavity and that in the pulp space. Sedgley et al. (13) showed that *E.faecalis* could be isolated from 5% and 55% of tongue samples, using culture and PCR methods, respectively. Viable enterococci are frequently found in fermented foods for raw consumption, such as cheese and meat, as well as in vegetables and olives (14,15). *E. faecium* is the main species found in meat, whereas *E.faecalis* predominates in cheese (14,15).

Fungi were reported in 2–21% of teeth with primary endodontic infections by researchers using culture and molecular methods (16). Fungi have been more commonly found in the root canals of obturated teeth with apical periodontitis, compared with necrotic root canals. The presence of yeasts in treatment-resistant cases ranges from 3 to 18% as shown by culture and PCR techniques (8,16). Candida species are known to be present in the oral cavity (16) and *C.albicans* is the most commonly isolated fungal species in the oral cavity (30–45% ) (16,17).

Recent studies using molecular methods have demonstrated that the microflora associated with endodontic infections are more diverse than was previously reported from studies using conventional culture methods (18,19). Molecular methods are typically faster, easier, and more precise than culturing methods. PCR is so sensitive that it can detect the presence of fewer than 10 bacteria in a sample. A major disadvantage of molecular methods is that organisms cannot be cultivated, so whether the cells are viable remains unknown (20).

The aim of this study was to investigate the presence of *E.faecalis* and *C.albicans* in endodontic infections using PCR.

## Material and Methods

Patients who were referred to Cukurova University Dental School for endodontic treatment or retreatment were included. Medical and dental histories were obtained from each patient. Age, gender, tooth type, pulp status, pain, history of previous pain, tenderness to percussion, pain on palpation, mobility, presence of a sinus, presence of swelling, history of previous and present antibiotic therapy, periapical radiolucency and radiographic findings with PAI (21) index were recorded ( Table 1). For necrotic teeth, an electric pulp test was conducted.

**Table 1:**
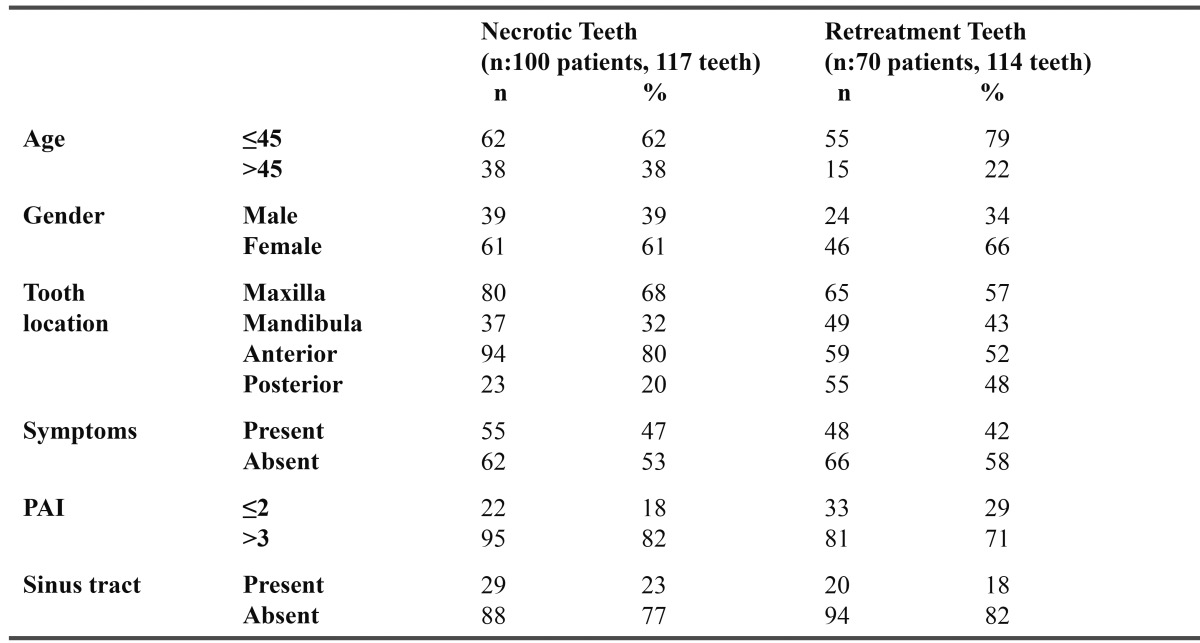
Characteristics of patients and necrotic teeth and retreatment teeth samples.

The study protocol describing specimen collection was approved by the Ethical Committee in Research of the Dental School of Cukurova University. Informed consent was obtained from all patients.

Microbial samples were obtained from 117 teeth of 100 patients with necrotic pulp tissues and 114 teeth of 70 patients with failed endodontic treatment. Control strains of *E.faecalis* (ATCC 97008) and *C.albicans* (ATCC 90028) were obtained from the culture collection at Refik Saydam Hıfzıssıhha Enstitüsü, Ankara, Turkey.

Root Canal Sampling

Each tooth was cleaned with pumice and isolated with a rubber dam. Teeth that could not be fully isolated with a rubber dam were excluded from the study. A gingival barrier (OpalDam Light Cured Gingival Barrier, Ultra-dent) was used between the teeth and the rubber dam for each case. The tooth and surrounding field were cleaned with 35% hydrogen peroxide (H2O2) and decontaminated with a 5% sodium hypochlorite (NaOCl) solution. After disinfection, the coronal restorations were removed. After completion of the endodontic access with a sterile high-speed carbide bur, the tooth, clamp, and adjacent rubber dam were once again disinfected with 5% NaOCl and then inactivated with sodium thiosulphate to avoid interference with the bacteriological sampling. For a control, a sterile cotton pellet was placed for 2 min in the pulp chamber to assess the efficacy of the disinfection procedure. Control pellet were taken to the microbiology laboratory for processing within 2 h. Thioglucolate medium samples were incubated at 37ºC for 24 h. If microbial growth was detected on the sterile control cotton pellet samples taken from the pulp chamber, the samples were disregarded.

For microbial sampling, No.15 Ktype file with the handle cut off was introduced to a level approximately 1 mm short of the tooth apex, based on diagnostic radiographs, with a discrete filing motion. If the root canal was dry, a small amount of sterile saline solution was introduced into the canal . Afterwards, three sterile paper were placed into the canal, with each left for 1 min for absorbing all the fluids present within them.These paper points were then transfered to cyrotubes containing TE buffer and immediately frozen at -20ºC.

Cases with failed endodontic treatments were sampled as follows. After plaque removal, isolation, and disinfection of the operative field as described aboved, coronal restorations were removed and the same disinfection protocol was used. Preexisting root canal fillings were removed using a Gates-Glidden drill and the apical material was retrived using Ktype files without the use of chemical solvents. Sterile saline solution was introduced into the canal to remove any remaning materials and to release the debris. The same procedure was used for the root canal sampling.

DNA Extraction 

DNA extraction of samples was performed using the QIAamp DNA mini kit (Qiagen, Valencia, CA) according to the manufacturer’s instructions. 100 μL’s of each clinical sample was used for DNA extraction. Briefly, samples were thawed to 37°C for 10 min and vortexed for 30 s. The microbial suspension was centrifugated for 10 min at 5000 g and the pellets were then resuspended in 180 μL ATL buffer (Qiagen). Afterwards, 20 μL proteinase K (20 mg/ml) was added and samples were incubated for 3 h at 56 °C. Next, 200 μL of ethanol was added and DNA was isolated by adding the lysate to the Qiagen columns as described by manufacturer. Finally, the total bacterial DNA was eluted with 200 μL AE buffer (Qiagen). DNA extracts were stored at -20°C. Reference DNA from target bacteria (*E.faecalis* ATCC strain 97008 and *C.albicans* ATCC strain 90028) were also extracted in the same manner. They served as controls for the primers used in this study. The DNA concentrations in clinical samples and the concentrations of the referance DNA were determined by spectrophotometer measurement of the absorbance at 260 nm.

DNA Amplification

A species–specific PCR assay with primers targeted to the 16S rRNA gene was done for *E.faecalis* and *C.albicans*. A pair of universal eubacterial primers that matches almost all bacterial 16S rRNA genes was used as a positive control for PCR reaction. It served to indicate the presence of bacteria in the clinical samples. The primers sequences of universal primer is F: 5’GAT TAG ATA CCC TGG TAG TCC AC-3’ and R: 5’-CCC GGG AAC GTA TTC ACC G-3’ (22). To identify enterococci species, enterococcal chromosomal DNA was amplified using the primers F: 5’-GTT TAT GCC GCA TGG CAT AAG AG-3’ and R: 5’-CCG TCA GGG GAC GTT CAG-3’ targeted to the *E.faecalis* 16S rRNA (23). Candidal chromosomal DNA was amplified using the primers F:5’-GCC GGT GAC GAC GCT CCA AGA GCT G-3’ and R: 5’-CCG TGT TCA ATT GGG TAT CTC AAG GTC-3’ (24). PCR reaction used to assess the occurrence of all target bacteria was performed with 50 µL of reaction mixture containing 0.5 μM of each specific primer, 2.5 mM MgCl2, 0.2 mM dNTP, 2.5 U Taq polymerase (Qiagen), and 10 µL extracted DNA. Amplification was carried out in a thermal cycler (Applied Biosystems 2720 Thermal Cycler, Singapore) with an initial denaturation step of 95°C for 2 min and followed by 36 cycles of a denaturation step at 95°C for 30 s and primer annealing step at 60°C for 1 m, an extension step at 72°C for 1 min, and a final step at 72°C for 2 min for Universal 16S r RNA primer. The temperature profile for *E.faecalis* included an initial denaturation step of 95°C for 15 min and followed by 35 cycles of a denaturation step at 94°C for 30 s and primer annealing step at 60°C for 1 m, an extension step at 72°C for 1 min, and a final step at 72°C for 2 min. The temperature profile for* C.albicans* included an initial denaturation step at 95°C for 15 min and followed by 35 cycles of a denaturation step at 95°C for 1 min and primer annealing step at 55°C for 30 s, an extension step at 72°C for 1 min, and a final step at 72°C for 10 min. ( Table 2). For negative control ultrapure water was used.

**Table 2:**
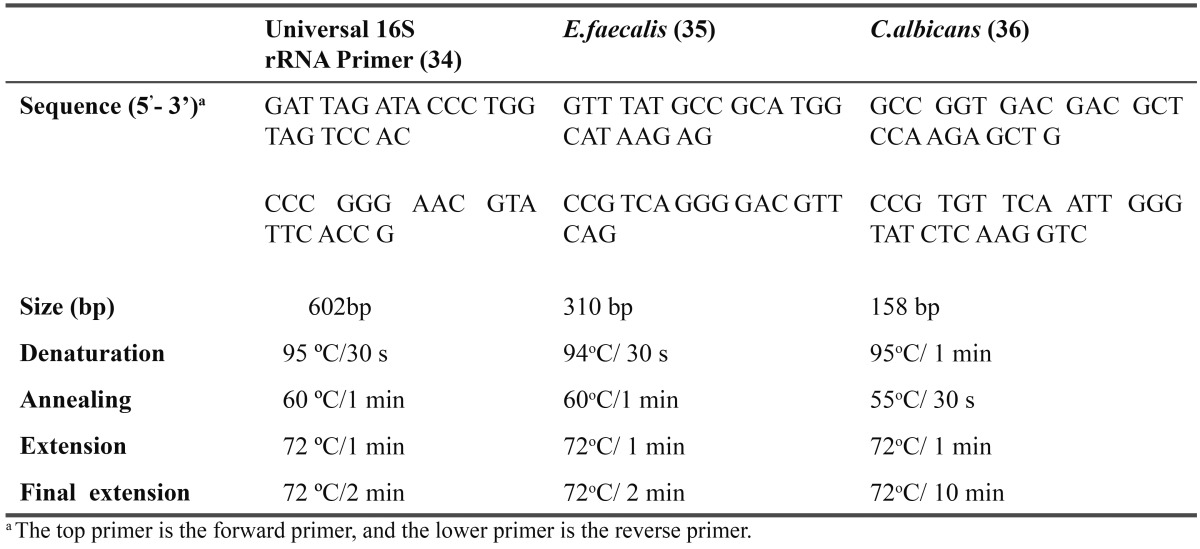
Oligonucleotide primer pairs used with PCR conditions.

PCR products were analyzed by 2% agarose gel (Seakem GTG agarose, FMC Bioproducts, Rockland, ME, USA) electrophoresis performed at 120 V in Tris-Borate EDTA buffer. The gels were stained with 0.5 µg/mL of ethidium bromide and visualized under ultraviolet light, and photographed with the Kodak Gel Logic 1500 Imaging System. We used a 100-bp DNA ladder digest (Invitrogen, Sao Paulo, Brasil) as a molecular weight marker.

The data collected were statistically analyzed using SPSS (ver. 12.0; SPSS Inc, Chicago, IL). Catagorical variables between the groups were analyzed using the chisquared test or the McNemar test. Results are presented as n (%). All reported pvalues are two-tailed.

## Results

**Figure 1:**
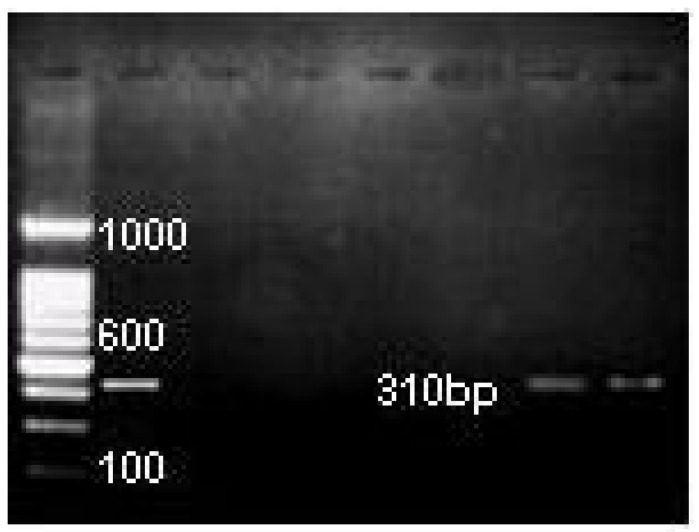
PCR images of E.faecalis in root canal samples (1 positive control, 2 negative control, 3-7 root canal samples).

**Figure 2:**
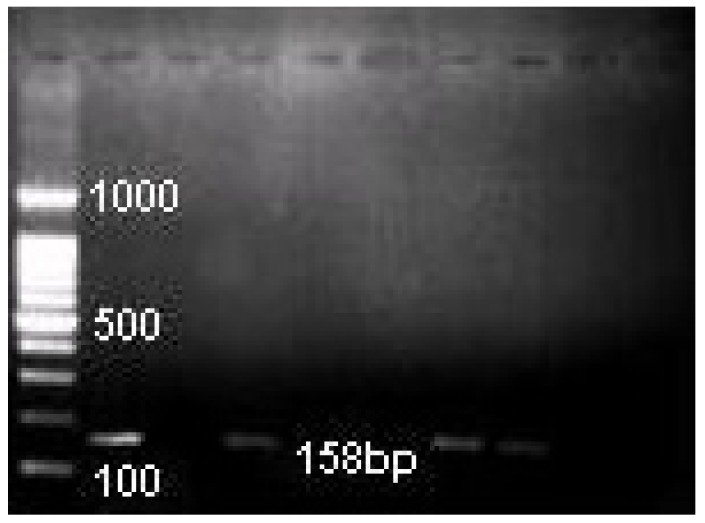
PCR images of C.albicans in root canal samples (1 positive control, 2 negative control, 3-9 root canal samples).

*E.faecalis* was identified in 19 (16%) of 117 necrotic canals and 11 (10%) of 114 retreated canals and *C.albicans* was identified in 23 (20%) and 13 (11%) necrotic and retreated canals, respectively by PCR analysis. (Fig. 1, Fig. 2). No significant difference was observed in the comparison of the prevalence of these species in necrotic and root-filled teeth (p > 0.05; chisquared test).

## Discussion

We have investigated the presence of *E.faecalis* and *C.albicans* in primary and secondary infected root canals. These microorganisms were found to be the most resistant species in the oral cavity to root canal treatment procedures, as shown in recent studies (2,16), and they can survive in the challenging conditions of the root canal ecosystem (3,27).

*E.faecalis* has been found in varying ratios in primary endodontic infections and root filled cases in previous studies (6). This wide diversity may be attributed to sample size, the quality of the initial treatment, the filling material, and different identification techniques (9,20). Geographical differences in these studies may influence the results. For example, in refractory cases, *E.faecalis* was detected in 77% of cases in a Brazilian population (6), 64% of South Korean patients (8), 47% of a German population (25), 56% of Lithuanian patients (3), 72% of Italian patients (26), and 30% of a North American population (27). On the other hand Ozbek et al. identified *E.faecalis* in 74.4% of root-filled teeth using real-time PCR east part of Turkey, which disagree with our results (28).

In recent years, PCR techniques have been increasingly used for the analysis of root canal flora (6-9). In PCR assays, it is not known whether the microorganisms detected can reproduce or survive (20). Molecular techniques can rapidly and accurately identify *Enterococcus* species with a higher sensitivity than can culturing from root canals. It may be expected that all teeth harbor microorganisms because periapical disease was present for all teeth. However, culturing and PCR showed a prevalence of microorganisms well below 100% (26). Microorganisms may be present in the apical ramification and go undetected because they are covered by remaining root canal-filling material and therefore not soaked up by the paper point (26).

The results of this study showed that *E.faecalis* rate in retreatment was significantly lower than the previous studies (8,28). Additionally, the *E.faecalis* prevalence was not statistically different between necrotic and re-treatment cases. It is noteworthy that all examined samples contained bacteria. These findings were confirmed by amplification with 16S rRNA universal primers, which generated the predicted amplicon for all cases. Consistent with our results, some studies analyzing the microflora of root filled teeth with apical periodontitis have shown lower rates than in necrotic root canal therapy cases (2,4). This may be caused by residual bacteria present in the accessory canals within the dentinal tubules, or it may be associated with the remnants of gutta perca, which may eventually prevent the sampling fluid from reaching the bacteria.

Another possible explanation may be the loss of bacteria during the sampling and cultivation procedures. In the study by Sassone et al. (1) and Ozbek et al. (29), samples were collected from the root canals using 15 Hedströen-type files and paper points. In this study, the samples were taken only by paper points after reaming, which might reduce the amount of bacteria found in the root canal. If *E.faecalis* can penetrate deeply into the dentinal tubules, paper points may not be sufficient to sample this microorganism. However, Peter et al. (30) suggested that the reaming sampling technique did not improve recovery rates.

Ravazzi et al. (14) demonstrated that young subjects with good oral hygiene and no caries or inflammatory oral diseases do not ordinarily harbor enterococci in their oral cavities. Differences in findings between the present study and the previously mentioned studies may also be due to different dietary intake of, for example, cheese, because it has been reported that viable enterococci occur frequently and in high numbers in cheese (14). In another study we investigated *E.faecalis* in 17 different kinds of cheese consumed in Turkey by culture method. We didn’t identify *E.faecalis* in any cheese samples (31).

The presence of yeast is more common in persistent infections after root canal preparations, probably due to contamination during treatment, or in cases of root-filled teeth with therapy-resistant periapical lesions. In the present study, the prevalence of *C.albicans* was not statistically different between necrotic and retreated root canals (p > 0.05).

Despite the lack of even minor evidence that Enterococci are responsible for refrectory periapical inflammation, the endodontic community has apparently accepted a causal connection as fact. Molecular techniques are superior for sampling low numbers of bacteria and identifying very fastidious species. For more significant research on endodontic diseases using molecular techniques, safeguards and standardized procedures need to be established. Future studies should evaluate resistance profiles of *C.albicans* and *E.faecalis* isolated from root canals to the antiseptic agents used in dentistry.

In conclusion, within the limitation of this study, the results showed that *C.albicans* and *E.faecalis* were associated with root-canal infections in primary and secondary infections. These microorganisms may be potent pathogens in apical lesions; however, the present study does not provide strong evidence to support this hypothesis. Additionally, PCR analyses of teeth with periapical lesions revealed that the *E.faecalis* percentage was lower than rates found in previous studies, whereas the *C.albicans* prevelance was consistent with previous results.
